# Evaluation of Glucocorticoid Receptor Function in COPD Lung Macrophages Using Beclomethasone-17-Monopropionate

**DOI:** 10.1371/journal.pone.0064257

**Published:** 2013-05-21

**Authors:** Jonathan Plumb, Laura Robinson, Simon Lea, Antonia Banyard, John Blaikley, David Ray, Andrea Bizzi, Giorgina Volpi, Fabrizio Facchinetti, Dave Singh

**Affiliations:** 1 National Institute for Health Research Translational Research Facility, Manchester Academic Health Science Centre, University Hospital of South Manchester Foundation Trust, University of Manchester, Manchester, United Kingdom; 2 School of Medicine, University of Manchester, Manchester, United Kingdom; 3 Chiesi Pharmaceuticals, Parma, Italy; Baylor College of Medicine, United States of America

## Abstract

Previous studies of glucocorticoid receptor (GR) function in COPD lung macrophages have used dexamethasone to evaluate inhibition of cytokine production. We have now used the clinically relevant corticosteroid beclomethasone-17-monopropionate (17-BMP) to assess GR function in COPD lung macrophages, and investigated the transactivation of glucocorticoid sensitive genes and GR phosphorylation in addition to cytokine production. Lung macrophages were purified from surgically acquired lung tissue, from patients with COPD, smokers, and non-smokers. The transactivation of glucocorticoid sensitive genes (FKBP51 and GILZ) by 17-BMP were analysed by polymerase chain reaction. 17-BMP suppression of LPS-induced TNFα, IL-6 and CXCL8 was measured by ELISA and GR phosphorylation was measured by immunohistochemistry and Western blot. 17-BMP reduced cytokine release in a concentration dependent manner, with >70% inhibition of all cytokines, and no difference between COPD patients and controls. Similarly, the transactivation of FKBP51 and GILZ, and GR phosphorylation was similar between COPD patients and controls. In this context, GR function in COPD lung macrophages is unaltered. 17-BMP effectively suppresses cytokine production in COPD lung macrophages.

## Introduction

Chronic obstructive pulmonary disease (COPD) is characterised by progressive airway inflammation [1]. Different cell types are involved in this inflammatory process, including lymphocytes, macrophages and neutrophils. The number of macrophages are increased in the lungs of COPD patients [1], [2], and also increase with disease severity [1]. Macrophages can promote inflammation through the production of cytokines, chemokines and proteases [3].

Inhaled corticosteroids (ICS) are the most widely used anti-inflammatory treatment for COPD. Corticosteroids bind to the cytoplasmic glucocorticoid receptor (GR); this complex translocates to the nucleus where it can exert anti-inflammatory effects by the transrepression of proinflammatory genes. Such transrepression appears to result from binding to, and inhibition of proinflammatory transcription factors, including nuclear factor kappa-light-chain-enhancer of activated B cells (NF-κB) [4]. Clinical trials have shown that ICS used in combination with long acting beta agonists improve lung function, exacerbation rates and health status [5], [6]. However, ICS do not completely suppress airway inflammation in COPD patients [1], [7], [8].

It is known that approximately half of the genes upregulated in lipopolysaccharide (LPS) stimulated healthy mouse alveolar macrophages are corticosteroid insensitive [9]. Similarly, we have observed that the effect of corticosteroid varies between cytokines secreted by LPS-stimulated alveolar macrophages from COPD patients and healthy subjects [10], [11]. The concentration of the neutrophil chemoattractant CXCL8 is increased in the airways of COPD patients, and we have observed that the production of this chemokine by alveolar macrophages is relatively insensitive to corticosteroids; this may be an important mechanism contributing to persistent neutrophilic inflammation in COPD that is incompletely suppressed by corticosteroids.

Earlier studies proposed that COPD macrophages are more corticosteroid insensitive compared to controls [12], [13]. However, in previous studies we have not confirmed these findings [10], [14], but observed that COPD and control macrophages both display intrinsic corticosteroid insensitivity that affects certain genes including neutrophil chemokines. The presence of greatly elevated numbers of macrophages in COPD lung [1], [2] increases the quantity of such corticosteroid insensitive proteins.

To further investigate whether GR function in COPD macrophages is altered compared to controls it is possible to study corticosteroid induced transcription of target genes that have glucocorticoid response elements (GREs) in their promoter regions [15], which is termed transactivation. GR function can also be studied by evaluation of its phosphorylation status; ser211 phosphorylation is essential for full GR activity [16], ser226 phosphorylation is associated with GR nuclear export [17] and ser203 phosphorylation is associated with a lack of GR nuclear accumulation and low levels of GR activity [18], [19]. The kinases that regulate GR phosphorylation include the mitogen activated protein kinases (MAPK) [20]. A range of inflammatory signals upregulate MAPK activity and p38 MAPK activation is increased in the lungs of COPD patients [21].

A limitation of previous studies using COPD alveolar macrophages is that dexamethasone was used [10], [11], [12], which is not an inhaled therapy for COPD. Beclomethasone-17-monopropionate (17-BMP) is the active metabolite of the ICS beclomethasone [22]. It is known that different, synthetic corticosteroids not only have different potencies [23], but that they also induce different GR conformations, and so result in distinct profiles of biological activity. Therefore, studies using dexamethasone may not be generalisable to other corticosteroids.

Previous studies of GR function in COPD alveolar macrophages focusing on cytokine production have produced conflicting results, either showing no difference between COPD and control cells [10], [11], [14], or reduced corticosteroid action in COPD compared to control cells [12], [13].We now apply direct measures of GR activation to further investigate whether GR function is reduced in COPD macrophages; we have measured ligand-dependent GR phosphorylation and transactivation of GR target genes. We also extend our previous observations regarding cytokine production by using 17-BMP, which is a clinically relevant corticosteroid.

## Methods

### Study subjects

One hundred and six patients undergoing surgical resection for suspected or confirmed lung cancer (see Table 1 for overall demographics, details of patients involved in each experiment are available in Table S1) were recruited for different experiments. Samples from subgroups of these patients were used for individual experiments, with numbers described in figure legends. COPD was diagnosed based on GOLD guidelines [24]. The majority of patients were GOLD stage II. Controls were either smokers (S) with normal lung function or lifelong non-smokers (NS).

**Table 1:** Subject Demographics.

	COPD	Smokers	Non-smokers
Subjects	54	33	19
Sex (F/M)	21/33	18/15	13/6
Age (yr)	66.3 (8.4)	64.8 (11.7)	56.9 (12)
FEV1 (L)	1.65 (0.5)	2.3 (0.7)	2.4 (0.4)
FEV1 % predicted	65.4 (13.3)	91.3 (14.9)	109 (32.05)
FVC (L)	2.96 (0.8)	3.1 (0.9)	3 (0.5)
FEV1:FVC ratio	57.9 (9.7)	79.2 (12.1)	83.8 (17.1)
Smoking history	53 (22.4)	37.1 (17.5)	0
Current/Ex-smoker	38/16	21/12	na
ICS	16	0	0

Data presented as mean (SD). Forced expiratory volume in 1 second (FEV1), Forced vital capacity (FVC), smoking history is pack years, inhaled corticosteroid (ICS), not applicable (na).

### Ethics statement

This research, patient information and consent forms were approved by the local research ethics committee (NRES Committee North West – Preston, NRES Committee North West – Greater Manchester South, NRES Committee North West – Greater Manchester East). All subjects gave written informed consent.

### Lung macrophage culture

Details of primary lung macrophage culture are provided in Text S1. Briefly lung macrophages were seeded on flat bottomed plates in growth media at a concentration of 1×10^5^ or 4×10^5^ macrophages per well in 96-well or 24-well plates respectively. Macrophages were stimulated for 24 h with LPS (1 µg/ml, serotype O26:B6, Sigma Aldrich, Poole, UK) after a 2 h pre-incubation with 17-BMP or dexamethasone at concentrations stated in the text. Experiments were performed in triplicate. Supernatants from 96 well plates were removed and stored at −80°C for analysis by ELISA, while cells in 24 well plates were lysed for RNA extraction for analysis of FK506-binding protein 51 (FKBP51) and glucocorticoid-induced leucine zipper (GILZ) expression or western blot analysis as described in the online supplement. Preliminary experiments (n = 6) showed macrophage preparations to be 88% positive for CD68.

### Glucocorticoid Receptor (GR) translocation assay protocol

The cell-based GR-translocation assay in Enzyme Fragment Complementation format developed by DiscoveRx (Fremont, CA) was employed to quantitatively measure GR nuclear translocation [25]. CHO-K1 PathHunterTM cells were incubated with increasing concentrations of 17-BMP and dexamethasone as described in Text S1.

### Immunohistochemistry

Tissue blocks were obtained from an area of the lung as far distal to the tumor as possible, then formalin fixed and paraffin embedded. Phosphorylated GR (pGR) was detected using rabbit anti-human pGR ser203, pGR ser211 and pGR ser226 primary antibodies as described in Text S1. At least 200 alveolar macrophages, defined as mononuclear cells with well represented cytoplasm present in the alveolar spaces and not attached to the alveolar walls, were counted per section. The number of immunopositive cells were calculated and presented as a percentage of the macrophages population.

### Statistical analysis

Full details of the statistics used are supplied in Text S1, briefly: ELISA data were treated parametrically with differences between 17-BMP and dexamethasone analysed using paired t-tests at each concentration. Real time PCR data was non-normally distributed. Friedman tests (non-parametric repeated measures ANOVA) were performed to analyse changes in gene expression within each group while Two-tailed Mann-Whitney tests were performed between patient groups at each drug concentration. The IC_50_ (concentration required to inhibit LPS effect by 50%) and EC_50_ values (concentration required to elicit 50% of the maximal effect) were calculated. GR translocation data, compound potencies, EC_50_ and 95% confidence limits (CL), were derived from a four-parameter non-linear iterative curve fitting analysis.

## Results

### The effects of 17-BMP on LPS-stimulated cytokines

#### Protein secretion

**Figure 1:**
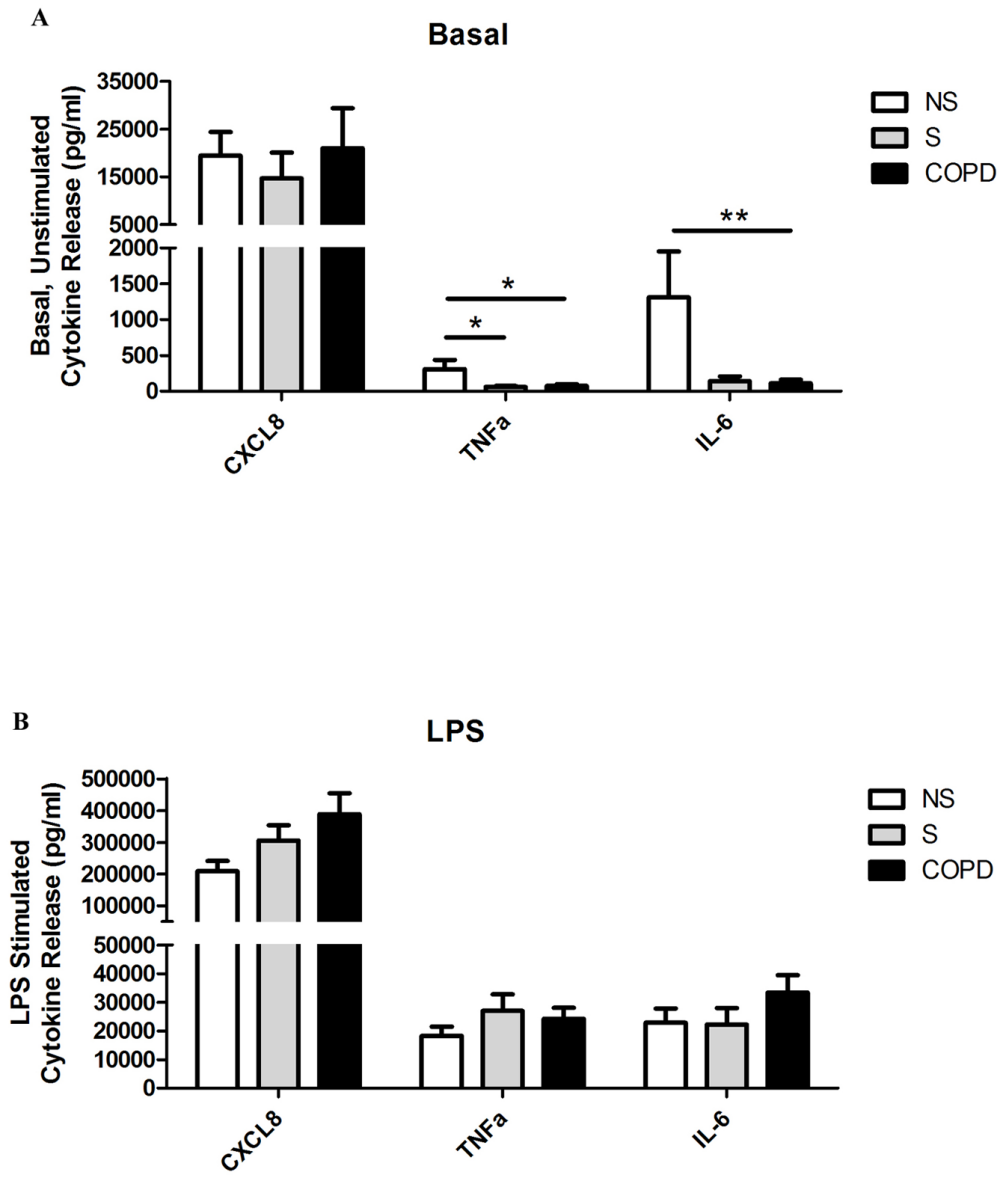
Baseline and LPS-stimulated characteristics of alveolar macrophage supernatants. Lung macrophages were incubated either with or without LPS (1 µg/ml) for 24 h with 17-BMP (0.01–100 nM). Data shown are mean ± SEM for CXCL8, TNFα and IL-6 ELISAs of baseline (A) and LPS-stimulated (B) supernatants. COPD (n = 25), S (n = 11) and NS (n = 8) shown. One-way ANOVAs were performed on all data sets, and when P<0.05 two-tailed t-tests were subsequently performed which are shown on the graph; * P<0.05, ** P<0.01.

Unstimulated levels of TNFα and IL-6 production from lung macrophages were significantly increased in NS (n = 8) compared to S (n = 11) and COPD (n = 25) patients (Fig. 1A). There was no difference between groups for unstimulated CXCL8 levels. LPS significantly increased the levels of all 3 cytokines. There were no differences between groups for any measured cytokine after LPS-stimulation (Fig. 1B). In the COPD patients, the cytokine production in current and ex-smokers was similar (data not shown).

**Figure 2:**
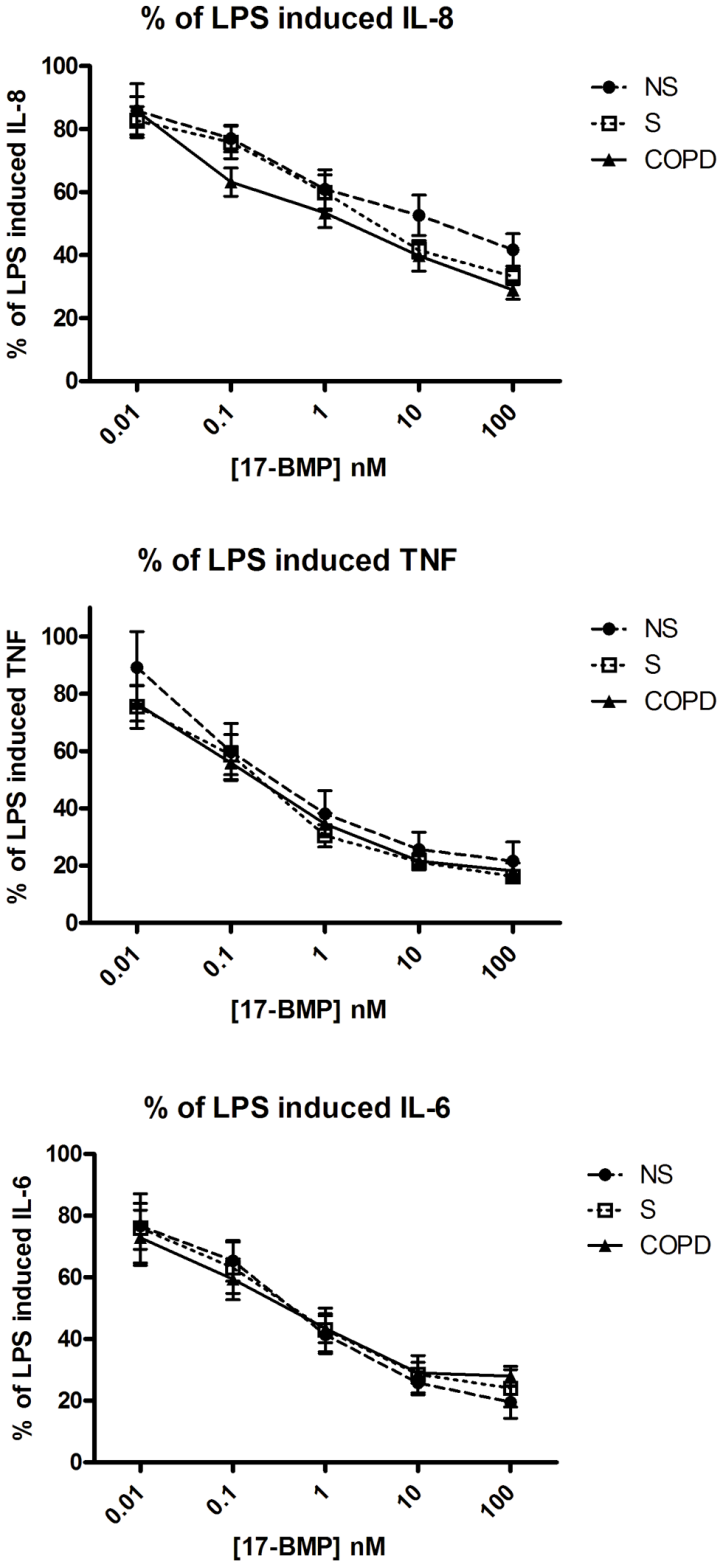
17-BMP inhibition of LPS-stimulated cytokine production. Lung macrophages were stimulated for 24 hours with LPS (1 µg/ml) after a 2 h pre-incubation with 17-BMP (0.01–100 nM). Graphs plotted as a percentage of LPS effect constrained to 100%, Data shown are mean ± SEM percentage inhibition for (A) CXCL8, (B) TNFα and (C) IL-6 ELISAs. COPD (n = 25), S (n = 11) and NS (n = 8) shown. One-way ANOVAs were performed between groups at each concentration; there was no difference between groups at any concentration.

17-BMP significantly inhibited LPS-stimulated CXCL8, TNFα and IL-6 release in a concentration dependent manner in all patient groups with maximal effect seen between 10 and 100 nM (Fig. 2); there were no differences between patient groups at any concentration (p>0.05 at each concentration). 17-BMP had a lower inhibitory effect on CXCL8 production compared to TNFα and IL-6 in each patient group. The IC_50_ and EC_50_ values for TNFα and IL-6 were below 1 nM in all patient groups, while values for CXCL8 were all over 1 nM (Table 2). Current smoking status, ICS use and gender did not influence 17-BMP effects on cytokine production (data not shown).

**Table 2:** Maximum inhibition, IC_50_ and EC_50_ for the effects of 17-BMP on LPS-stimulated macrophage cytokine production.

	Maximum % Inhibition				IC_50_ (nM)			EC_50_ (nM)		
	NS	S	COPD	p [A]	NS	S	COPD	NS	S	COPD
**CXCL8**	58.3 (14.6)	66.8 (8.4)	71.0 (14.8)	0.67	16.65	4.55	2.04	0.2	0.1	0.09
**TNFa**	78.4 (18.9)	85.8 (7.1)	81.7 (12.8)	0.08	0.5	0.21	0.21	0.08	0.08	0.06
**IL-6**	80.5 (14.7)	76.0 (18.0)	72.0 (15.3)	0.41	0.49	0.46	0.36	0.1	0.08	0.03
**p[B]**	0.0004	0.0156	0.0023							

The mean (SD) percentage inhibition at 100 nM (maximal inhibition). One-way ANOVA were performed between patient groups (p values in column [A]) and between cytokines (p value in row [B]). IC_50_ (concentration required to inhibit LPS effect by 50%) and EC_50_ values (concentration required to elicit 50% of the maximal effect) of 17-BMP for each cytokine are shown. COPD (n = 25), S (n = 11) and NS (n = 8).

#### mRNA levels

**Figure 3:**
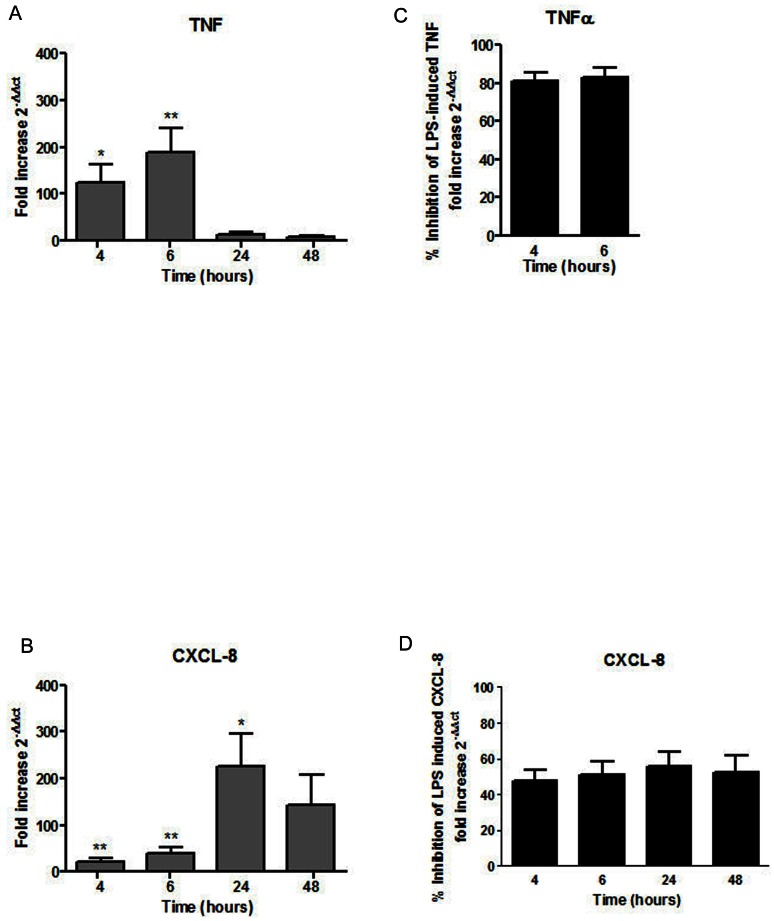
17-BMP inhibition of LPS-stimulated cytokine transcription. Lung macrophages (COPD n = 4 and S n = 3) were treated with LPS (1 ug/ml) for 4, 6, 24 and 48 h and CXCL8 and TNFα mRNA levels were measured. Data are presented as fold change over time compared to time matched unstimulated controls for (A) TNFα and (B) CXCL8. The effect of 17-BMP on (C) TNFα transcription and (D) CXCL8 transcription was also analysed and presented as % Inhibition of LPS-induced TNF fold increase. * = significantly above time matched unstimulated control (p<0.05) ** = significantly above time matched unstimulated control (p<0.01).

As we observed that 17-BMP had a reduced inhibitory effect on CXCL8 protein secretion compared to TNFα, we now investigated whether this difference was also apparent at the level of gene transcription. As there was no difference between COPD patients and controls for the effects of 17-BMP, we used pooled data from COPD patients and S for this experiment. Lung macrophages from COPD patients (n = 4) and S (n = 3) were treated with LPS (1 ug/ml) for 4, 6, 24 and 48 h and CXCL8 and TNFα mRNA levels were measured (Fig. 3). TNFα mRNA levels peaked at 6 h and returned to baseline levels by 24 h (Fig. 3A). In contrast, CXCL8 mRNA levels peaked after 24 h and then declined but had not returned to baseline by 48 h (Fig. 3B). LPS-induced CXCL8 and TNFα transcripts were inhibited by 17-BMP (100 nM) (Fig. 3C&D) with TNFα (approximate mean inhibition 80%) showing significantly greater response to 17-BMP than CXCL8 (approximate mean inhibition 50%) at 4 and 6 h (p = 0.01 and 0.002 respectively).

### Comparison of 17-BMP and dexamethasone

As previous studies of the effects of corticosteroids on lung macrophages have used dexamethasone, we compared the potency of 17-BMP (0.01–1000 nM) with dexamethasone (0.01–1000 nM) on COPD lung macrophages (n = 9).

**Figure 4:**
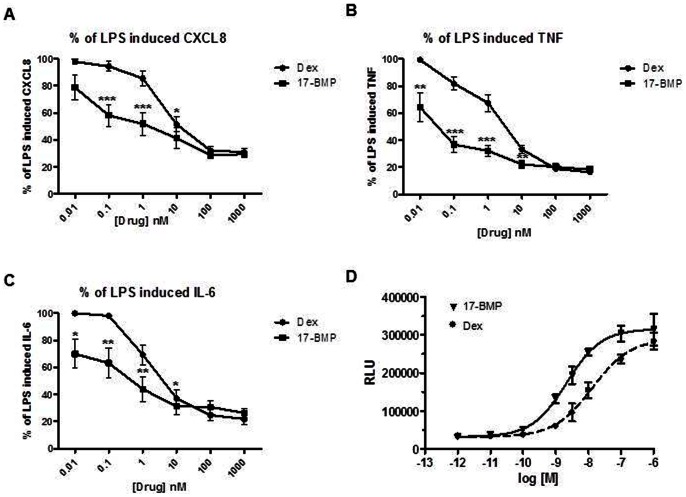
Comparative potency of 17-BMP and dexamethasone on LPS-stimulated cytokine production and nuclear translocation. Lung macrophages (COPD n = 9) were stimulated for 24 hours with LPS (1 µg/ml) after a 2 h pre-incubation with 17-BMP (0.01–100 nM) or dexamethasone (0.01–1000 nM). Data shown are mean ± SEM percentage of LPS effect constrained to 100% for the production of (A) CXCL8, (B) TNFα and (C) IL-6 ELISAs. Two-tailed paired t-tests were performed at each concentration; * P<0.05, ** P<0.01, *** P<0.001. (D) Concentration-response curves in CHO-K1 PathHunterTM cells incubated with 17-BMP or dexamethasone (1×10^−12^–1×10^−5^ M) for 3 h. Data shown are mean ± SD of a representative experiment performed in triplicate.

17-BMP inhibited LPS-stimulated CXCL8, TNFα and IL-6 more potently than dexamethasone at concentrations from 0.01–10 nM for IL-6 and TNFα and 0.1–1 nM for CXCL8 (p<0.05 at these concentrations) (Fig. 4A–C). There were no differences between drugs at higher concentrations. The EC_50_ values of 17-BMP for IL-6, TNFα and CXCL8 were 0.05 nM, 0.01 nM and 0.1 nM respectively. The EC_50_ values for dexamethasone were higher, at 1.7 nM, 1.7 nM and 4.6 nM respectively.

17-BMP and dexamethasone (1×10^−12^–1×10^−5^ M) were used to induce GR nuclear translocation in CHO-K1 PathHunterTM cells; the EC50 values were 2.0 nM (95% CL. 1.3–3.2 nM) and 13.2 nM (95% CL. 8.4–20.7 nM) respectively. Both agonists exhibited concentration–response curves that attained similar maximum effects (Fig. 4D).

### The effects of 17-BMP on FKBP51 and GILZ gene expression

**Figure 5:**
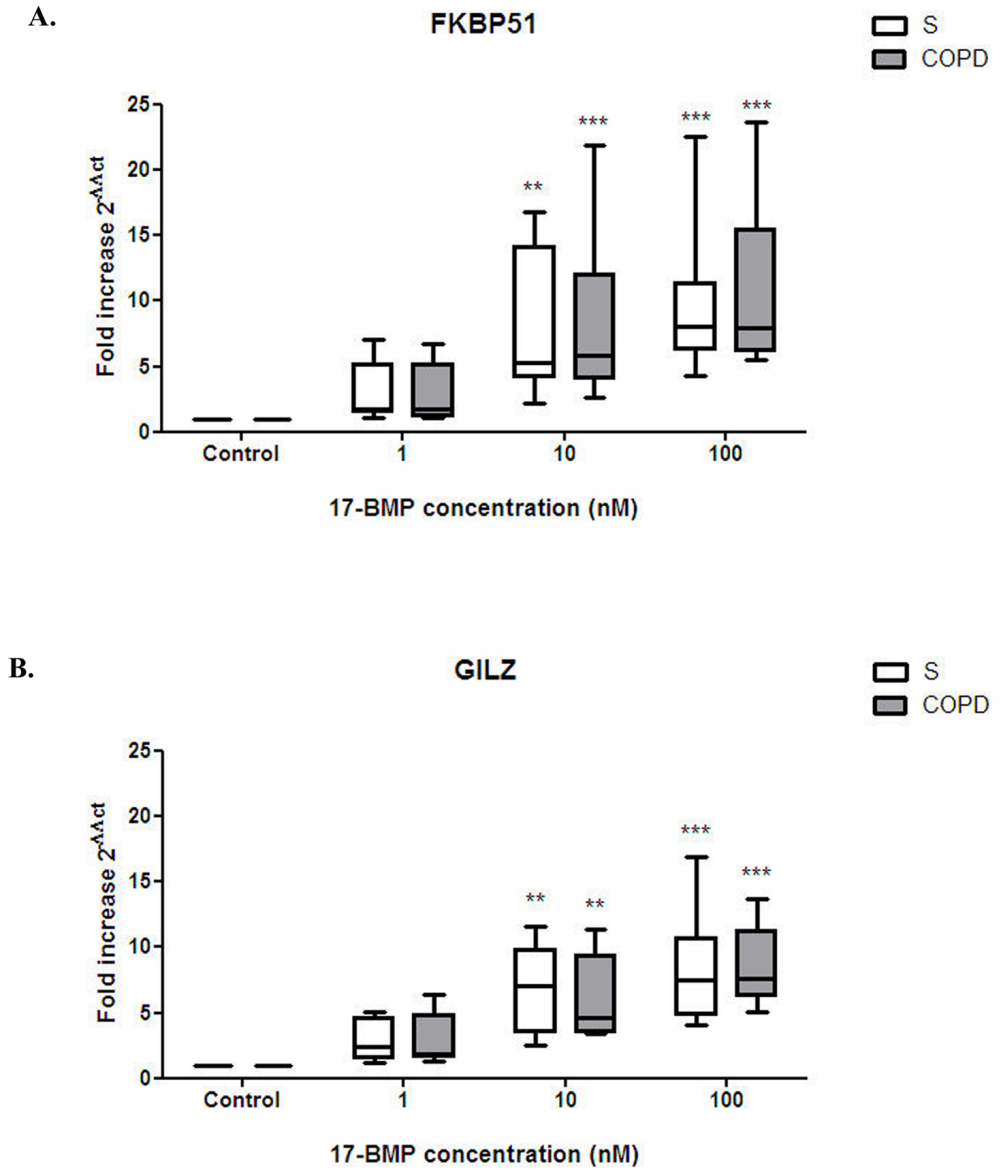
17-BMP evokes upregulation of the GR dependent genes FKBP51 and GILZ. Lung macrophages from smoking controls (n = 7) and COPD patients (n = 7) were treated with 17-BMP (1–100 nM) or media alone for 24 h. RNA was extracted for PCR analysis of (A) FKBP51 and (B) GILZ expression. Data shown are median + range of fold increase of gene expression above controls. Box represents the 1^st^ and 3^rd^ quartile of the data set. Friedman Tests followed by Dunn's multiple comparison tests were performed to determine upregulation compared to baseline: ** P<0.01, *** P<0.005. Two-tailed Mann-Whitney tests were performed between patient groups at each drug concentration; there was no difference between groups.

Lung macrophages from 7 COPD patients and 7 S were treated with 17-BMP (1–100 nM) or media alone for 24 hrs. 17-BMP significantly upregulated expression of both FKBP51 and GILZ in both patient groups (p<0.01). There were no differences in the fold increase of these genes for each concentration of 17-BMP between COPD patients and S (Fig. 5).

### GR phosphorylation

**Figure 6:**
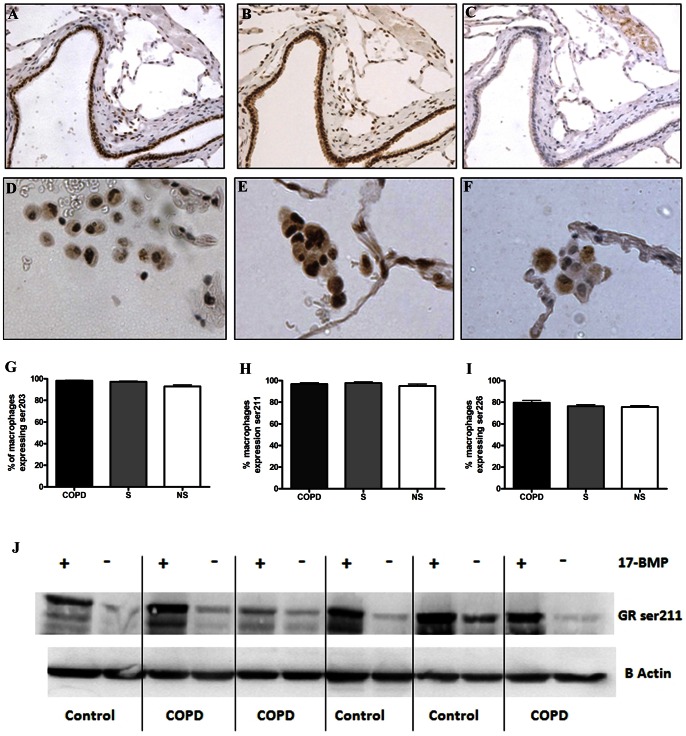
Glucocorticoid receptor phosphorylation. GR phosphorylation status was examined *in situ* via immunohistochemistry in COPD (n = 16), S (n = 9) and NS (n = 11). (A–C) GR phosphorylation at ser203, ser211 and ser226 on serial sections through the same small airway. (D–I) GR was phosphorylated at ser203, ser211 and ser226 within alveolar macrophages from all groups. Data are presented as mean (SEM). (J) Examination of GR phosphorylation was examined *ex vivo* via western blotting. Lung macrophages were treated for 1 h with 1 µM 17-BMP and cell lysates analysed for ser211 phosphorylation. Blot representative of samples from COPD (n = 6) and smoking controls (n = 6). Different subjects were used in the immunohistochemical analysis and the *ex vivo* analysis.

The phosphorylation status of GR in peripheral lung tissue was analysed by immunohistochemistry using samples from COPD patients (n = 16), S (n = 9) and NS (n = 11); small airway epithelium and cells within the submucosa displayed GR that was phosphorylated at ser203 and 211 in all 3 subject groups, but there was no evidence of phosphorylation at ser226 (Fig. 6A–C). In contrast alveolar macrophages expressed phosphorylation of GR at ser203, ser211 and ser226 (Fig. 6D–F). GR was found to be phosphorylated in a high proportion of macrophages in both COPD patients and controls. There was no difference in ser211 and ser226 phosphorylation in alveolar macrophages between groups, with only a minor increase in ser203 expression in alveolar macrophages from COPD patients and S compared to NS (p = 0.0002, Fig. 6G–H).

Lung macrophages from COPD (n = 6) patients and S (n = 6) displayed similar phosphorylation of GR at ser211 following incubation with 17-BMP (1000 nM) for 1 h *in vitro* (Fig. 6J).

## Discussion

We observed no difference in GR function in lung macrophages from COPD patients compared to controls. The effects of 17-BMP on LPS-stimulated cytokine production and the transactivation of FKBP51 and GILZ in COPD and control lung macrophages were similar. Furthermore, GR phosphorylation in COPD and control lung macrophages was similar, both in immunohistochemistry studies and functional experiments using 17-BMP. In contrast to some previous reports [12], [13], these findings demonstrate that the sensitivity of lung macrophages to the effects of 17-BMP is not changed in COPD patients compared to controls.

17-BMP potently inhibited LPS-stimulated TNFα and IL-6 production, with low EC_50_ values observed for these cytokines. In contrast, 17-BMP had less inhibitory effect on CXCL8 production compared to both TNFα and IL-6; this is consistent with our previous results investigating the effects of dexamethasone on alveolar macrophages [10], [11], [14], and similar to data from human airway smooth muscle cells [26]. We also observed that the effect of 17-BMP on CXCL8 transcription was reduced compared to TNFα. Therefore, the effects of 17-BMP appear to differ between target cytokines and chemokines, with reduced effects seen on CXCL8. This is consistent with previous data showing that certain transcriptional responses in macrophages are not responsive to corticosteroids [9].

We examined the corticosteroid transactivation of two genes that contain GREs in order to investigate a possible change in GR function in COPD macrophages. We studied FKBP51 and GILZ, which are also both modulators of corticosteroid activity in inflammation. FKBP51 expression is increased by corticosteroids and the regulation of FKBP51 levels represents a possible feedback mechanism for inhibiting prolonged corticosteroid responses [27]. Increased expression of FKBP51 negatively modulates translocation and activation of GR [28], [29]. GILZ was originally discovered in studies aimed at characterizing genes targeted by dexamethasone and subsequently gained a distinguished reputation within the critical mediators of anti-inflammatory glucocorticoid effects as it interacts with several relevant pathways, including NF-κB, AP-1, Raf-1, and Ras [30]. We found that 17-BMP elicited a similar stimulatory effect on mRNA levels of FKBP51 and GLIZ in both COPD and S lung macrophages, arguing against loss of ability of ligand bound GR to exert effects on gene transcription in these cells.

Interestingly, while inhibition of TNFα and IL-6 occurred at subnanomolar concentrations, transactivation of GILZ and FKBP51 occurred at higher concentrations. This is consistent with the notion that transrepression, which is associated with monomeric GR activation, occurs at lower concentrations than transactivation, which is mediated by GR homodimers [31].

GR is a phosphoprotein containing multiple potential sites for phosphorylation, including ser203, ser211 and ser226. Altered GR phosphorylation status can affect a range of GR functions, including DNA binding and transactivation potential, hsp90 interactions, subcellular localization and nuclear-cytoplasmic shuttling [32]. This suggests that GR phosphorylation may be one of the molecular mechanisms of corticosteroid resistance. MAPKs can induce N-terminal phosphorylation of GR [33]; p38 activation has been implicated in ser211 phosphorylation [34], while c-Jun N-terminal kinase (JNK) activation induces ser226 phosphorylation leading to increased GR nuclear export [35].

Immunohistochemistry data presented here shows little or no difference in GR phosphorylation status in COPD macrophages compared to controls. We also demonstrate the hyperphosphorylation of GR *in vitro* at ser211 after exposure to 17-BMP is rapid and similar in lung macrophages from both COPD patients and controls; this indicates similar GR activation in both groups.

The high degree of phosphorylation of lung macrophages at ser203, ser211 and ser226 indicates that each alveolar macrophage is likely to contain GR proteins in different phosphorylation states, compatible with a dynamic process of GR phosphorylation and shuttling between the cytoplasm and nucleus under the control of intra- and extra-cellular signals [32]. It does not appear that this process is disrupted or altered in COPD patients.

The airways of patients with COPD are often colonised with bacteria, making LPS stimulation a physiologically relevant model to study macrophage pharmacology. We have used this model in previous studies of patients with COPD, with similar results for the effects of corticosteroids on CXCL8 [10], [11]. The transcription of CXCL8 is usually repressed by proteins such as the NF-κB repressing factor (NRF) [36]. The production of CXCL8 requires loss of repression coupled with transcriptional activation by NF-κB and AP-1. Furthermore, maximal CXCL8 levels occur when p38-MAPK activation causes post-transcriptional mRNA stabilisation [37]. Corticosteroids can recruit co-repressors and histone deacetylases to form complexes with the GR that suppress the actions of NF-κB – histone acetyltransferase – co-activator complexes at the promoter regions of inflammatory genes [38]. Corticosteroid efficacy is dependent on a range of factors, including the availability and activity of co-repressors [38]. The reduced effect of corticosteroids on CXCL8 production from lung macrophages may be due to decreased CXCL8 repressor activity. Moreover, GR activation can be directly opposed by a high degree of NF-κB activation [36], which appears to be the case for LPS-stimulated alveolar macrophages [36], [39].

In the current study, the transcription of CXCL8 followed a different time-course to TNFα after stimulation with LPS, with a later peak at 24 hours compared to 6 hours respectively. These findings further demonstrate the regulation of transcription of CXCL8 differs from other cytokines.

The levels of CXCL8 protein are known to be increased in the lungs of COPD patients [40], [41], [42] and are associated with the rate of disease progression [43]. We observed that CXCL8 production from unstimulated and LPS-stimulated lung macrophages was similar in COPD patients compared to S and NS. There are increased numbers of macrophages in the lungs of COPD patients compared to controls [1], [2]. However, ex-vivo cell cultures do not take these differences into account, as the same numbers of macrophages are placed into each culture well. It is therefore possible that the increased number of macrophages contributes to the raised CXCL8 levels in the lungs of COPD patients. Additionally, other cell types such as epithelial cells also contribute to overall CXCL8 levels.

17-BMP partially suppressed CXCL8 production and potently suppressed TNFα and IL-6 production from lung macrophages. Inhaler devices that use particle sizes <2 µm enhance the fraction delivered to the peripheral airways [44]. Our data suggests that 17-BMP delivered as extrafine particles to the peripheral airways in COPD patients can effectively exert anti-inflammatory effects on alveolar macrophages, although the magnitude of effect varies between inflammatory mediators.

Previous studies using COPD lung macrophages have used dexamethasone to study corticosteroid insensitivity [10], [11]. However, dexamethasone is not an inhaled therapy. Furthermore, dexamethasone has a 10 fold higher IC_50_ value compared to beclomethasone for suppression of T cell cytokine production [45]. Similarly, we now demonstrate EC50 values that are at least 10 fold higher for dexamethasone compared to 17 BMP for lung macrophage cytokine production. 17-BMP was at least 100 fold more potent in eliciting GR nuclear translocation compared with dexamethasone in CHO cells. These observations suggest a weaker relative affinity of dexamethasone for GR, or differences in the interactions between these corticosteroids and GR, perhaps involving mechanisms modulating distinct aspects of receptor function, such as interactions with co-regulators [19].

The current study used macrophages from lung resections. This is a commonly used and accepted method [12], [46], [47]. An alternative is to obtain alveolar macrophages from bronchoscopy. Despite the different sources of macrophages, our studies using either lung resections or bronchoscopies have not shown corticosteroid insensitivity in COPD macrophages compared to controls [10], [11], and in all these experiments we have observed that CXCL8 was less corticosteroid sensitive than other cytokines.

In summary, we observed no loss of GR function in COPD lung macrophages compared to controls. Our data suggests that the effective delivery of 17-BMP to the distal airways is capable of suppressing the production of pro-inflammatory cytokines by COPD lung macrophages.

## Supporting Information

Table S1 **Subject demography.** Data shown as Mean (SD). Forced expiratory volume in 1 second (FEV1), Forced vital capacity (FVC), smoking history is pack years, inhaled corticosteroid (ICS), not applicable (na).
(DOC)

Text S1 **Detailed description of the materials and methods utilised in; lung macrophage culture, the glucocorticoid receptor (GR) translocation assay, RNA extraction and PCR, immunohistochemistry, western blotting and statistical analysis.**
(DOCX)
