# Supplementary material for: Evaluation of Glucocorticoid Receptor Function in COPD Lung Macrophages Using Beclomethasone-17-Monopropionate
Source: PLoS One. 2013 May 21;8(5):e64257. doi: 10.1371/journal.pone.0064257 (PMC3660317; doi:10.1371/journal.pone.0064257)
Supplement: Table S1 — Subject demography. Data shown as Mean (SD). Forced expiratory volume in 1 second (FEV1), Forced vital capacity (FVC), smoking history is pack years, inhaled corticosteroid (ICS), not applicable (na). (DOC) [file pone.0064257.s001.doc]

|  | **Cytokine Measurements** | | | **PCR** | | **Immunohistochemistry** | | | **Western blot** | |
| --- | --- | --- | --- | --- | --- | --- | --- | --- | --- | --- |
|  | **NS** | **S** | **COPD** | **S** | **COPD** | **NS** | **S** | **COPD** | **S** | **COPD** |
| **n** | 8 | 11 | 25 | 7 | 7 | 11 | 9 | 16 | 6 | 6 |
| Gold Stage I | n/a | n/a | 3 | n/a | 2 | n/a | n/a | 0 | n/a | 0 |
| Gold Stage II | n/a | n/a | 17 | n/a | 4 | n/a | n/a | 14 | n/a | 6 |
| Gold Stage III | n/a | n/a | 5 | n/a | 1 | n/a | n/a | 2 | n/a | 0 |
| **Age (yrs)** | 61.6 (7.0) | 64.0 (10.1) | 64.8 (9.8) | 73.6 (5.2) | 70.1 (6.9) | 53.5 (14) | 58.6 (12.8) | 65.1 (6.1) | 65.4 (18) | 69.6 (8) |
| **Sex (M / F)** | 0 / 8 | 7 / 4 | 18 / 7 | 3 / 4 | 6 / 1 | 6 / 5 | 3 / 6 | 6 / 10 | 2 / 4 | 3 / 3 |
| **FEV1 (L)** | 2.24 (0.2) | 2.60 (0.9) | 1.79 (0.6) | 2.05 (0.5) | 1.81 (0.4) | 2.6 (0.5) | 2.3 (0.7) | 1.04 (0.3) | 2.3 (0.6) | 1.5 (0.3) |
| **FEV1 % Predicted** | 111.4 (32.7) | 94.8 (15.6) | 62.9 (13.2) | 95.8 (20.0) | 73.4 (16.8) | 106.2 ( 34.1) | 87.4 (10.2) | 61.5 (7.6) | 89.6 (13.4) | 68.4 (7.4) |
| **FVC (L)** | 2.84 (0.3) | 3.42 (1.2) | 3.19 (0.8) | 2.74 (0.8) | 3.20 (0.8) | 3.2 (0.7) | 3 (0.8) | 2.5 (0.6) | 3.4 (0.8) | 2.9 (0.7) |
| **FEV1/FVC Ratio (%)** | 89.5 (21.9) | 84.3 (18.9) | 57.1 (10.4) | 75.9 (5.7) | 58.3 (11.7) | 77 (4.9) | 82.1 (8) | 61 (9.4) | 71.2 (3.4) | 52.9 (4.5) |
| **Pack Year History** | 0 | 47.2 (18.6) | 48.4 (19.1) | 27.7 (10.3) | 51.9 (23.4) | 0 | 58.6 | 56.6 | 29 (12.5) | 65 (31) |
| **Smoking Status (Current / Ex)** | n/a | 10 / 1 | 17 / 8 | 0 / 7 | 0 / 7 | n/a | 9 / 0 | 16 / 0 | 2 / 4 | 5 / 1 |
| **ICS users** | 0 | 0 | 12 | 0 | 1 | 0 | 0 | 3 | 0 | 2 |

**Table S1:** Subject demography. Data shown as Mean (SD).
