# Supplementary material for: Evaluation of Glucocorticoid Receptor Function in COPD Lung Macrophages Using Beclomethasone-17-Monopropionate
Source: PLoS One. 2013 May 21;8(5):e64257. doi: 10.1371/journal.pone.0064257 (PMC3660317; doi:10.1371/journal.pone.0064257)
Supplement: Text S1 — Detailed description of the materials and methods utilised in; lung macrophage culture, the glucocorticoid receptor (GR) translocation assay, RNA extraction and PCR, immunohistochemistry, western blotting and statistical analysis. (DOCX) [file pone.0064257.s002.docx]

**Macrophage culture**

Areas of lung distant from the tumour were perfused with 0.1M NaCl. The resulting cell suspension was centrifuged (400g, for 10 minutes, at room temperature) and the cell pellet resuspended in RPMI-1640. The cells were layered over a Ficoll-Paque (GE Healthcare, Buckinghamshire, UK) gradient. The mononuclear cells at the Ficoll interface were extracted, washed and resuspended in complete media: RPMI-1640 (Sigma-Aldrich, Dorset, UK) containing 10% v/v foetal calf serum (FCS; Invitrogen, Paisely, UK); 2mM L-Glutamine (Invitrogen); 100U/ml penicillin and 100µg/ml streptomycin (Sigma-Aldrich). Cells were counted using trypan blue exclusion and seeded as described in methods. Lung macrophages were seeded on flat bottomed plates in growth media at a concentration of 1x10^5^ macrophages per well of a 96-well plate for experiments measuring supernatant cytokine levels or 4x10^5^ macrophages per well of a 24-well plate for experiments measuring gene expression levels. Cells were incubated overnight at 37ºC, under a humidified atmosphere containing 5% CO2, to allow lung macrophages to adhere to the plate. Non-adherent cells were then removed by a change of media. Lung macrophages were stimulated for 24hrs with LPS (1µg/ml) after a 2hr pre-incubation with 17-BMP or dexamethasone at concentrations stated in the results text. ELISAs using the supernatant were performed for CXCL8, TNFα and interleukin-6 (IL-6), as per the manufacturers’ instructions (R&D Systems, Abingdon, UK). After the removal of supernatants, cell pellets were lysed in Trizol (Invitrogen) for RNA extraction and PCR analysis for FKBP51 and GILZ expression as described in the on-line supplement. Preliminary data (n=6) has shown macrophage preparations to be 88% positive for CD68.

**Glucocorticoid Receptor (GR) translocation assay protocol**

The cell-based GR-translocation assay in Enzyme Fragment Complementation (EFC) format developed by DiscoveRx (Fremont, CA) was employed to quantitatively measure GR nuclear translocation (Fung et al., 2006). Chinese Hamster Ovary-K1 (CHO-K1) cells stably expressing NLS-enzyme acceptor fragment (EA) of β-gal and GR-enzyme donor (ED) fragment of β-gal (herein referred to as CHO-K1 PathHunterTM cells) were maintained in F12 medium (Invitrogen, Carlsbad, CA) at 37°C under a humidified atmosphere containing 5% CO2. The medium contained 10% FBS, 2mM L-glutamine, 50U/ml penicillin 50µg/ml streptomycin, and 250µg/ml hygromycin and 500µg/ml G418 (Invitrogen). CHO-K1 PathHunterTM cells (1x105 cells per well of a 48 well plates) were incubated with increasing concentrations of 17-BMP, beclometasone dipropionate (BDP) and dexamethasone (1x10^-12^ – 1x10^-5^ M) for 3hrs at 37ºC. β-gal enzyme activity was detected by incubating cells (1hr at room temperature) with the PathHunter Detection Kit containing cell membrane permeabilizing reagent and β-gal substrate (DiscoveRx, Fremont, CA). Luminescence was detected using a CENTRO LB 960 microplate reader (Berthold Technologies, Oak Ridge, TN).

**RNA extraction and PCR**

Total RNA was purified from cell lysates using RNeasy kits (Qiagen, Crawley, UK) according to manufactuers’ instructions. DNA contamination was prevented by on-column addition of DNase (Qiagen, Crawley, UK) according to manufactures’ instructions. TaqMan reverse transcription- PCR (RT-PCR) was performed in triplicate on RNA from lung macrophages. 30ng of total RNA was reacted with VersoTM 1-step QRT-PCR kit (Thermo Scientific, Surry, UK) in 25µl reactions containing premade ABI Taqman gene expression assays for either TNFα (Catalogue no: Hs001113624_ml Applied Biosystems, Warrington, UK), CXCL8 (Catalogue no: Hs00174103_ml, Applied Biosystems), FKBP51 (Catalogue no: Hs00188025_m1, Applied Biosystems,) or GILZ (Catalogue no: Hs00608272_m1, Applied Biosystems) and the endogenous control was glyceraldehyde-3phosphate dehydrogenase (GAPDH) (Catalogue no: 4352934E, Applied Biosystems). Controls without RT-enzyme showed there was no genomic DNA amplification. Thermal cycling was carried out on a Stratagene MX3005P (Agilent Technologies, West Lothian, UK). Relative expression levels were determined using the ΔΔCt method with untreated cells as the calibrator

**Immunohistochemistry**

Tissue blocks were obtained from an area of the lung as far distal to the tumor as possible, then formalin fixed and paraffin embedded. Phosphorylated GR (pGR) was detected using either rabbit anti-human pGR ser203 (Abcam, Cambridge, UK), pGR ser211 (New England Biolabs, Hitchin, UK) or pGR ser226 (Abcam, Cambridge, UK) primary antibodies. Heat induced epitope retrieval (HIER) was performed by microwaving for 20 min at 800W in a 1mM trisodium citrate buffer pH6. Primary antibody was applied overnight at 4°C. Targets were detected using biotinylated Ig(G) secondary antibodies (Vector Labs, Peterborough, UK) in conjunction with an avidin-biotin peroxidase complex and 3,3’-diaminobenzidine (DAB) substrate (both Vector Labs). Sections were counterstained with Meyer's haemotoxylin (Sigma, Poole, UK).

**Western Blot Analysis**

Cells (4 × 106) were lysed in RIPA buffer [10 mM Tris-HCl, pH 7.4, 150 mM NaCl, 1 mM EDTA, 1% Nonidet P-40, 0.25%] containing phosphatase (Sigma Aldrich, Dorset, UK) and protease inhibitors (Calbiochem, Nottingham, UK). Cell lysates diluted in sample buffer [62.5 mM Tris, 10% glycerol, 1% SDS, 1% β-mercaptoethanol, and 0.01% bromphenol blue, pH 6.8] were electrophoresed on SDS-polyacrylamide gels (10%) and transferred to Hy-bond ECL membranes (Whatman, Kent, UK). Membranes were incubated with blocking buffer [5% dried milk in Tris-buffered saline containing 0.1% Tween 20 (TBS/Tween 20)] for 1hour at room temperature and then incubated with primary antibodies (diluted in blocking buffer) at 4 °C overnight. After washing in TBS/Tween 20, the membranes were incubated for 1hour with a peroxidase-conjugated secondary antibody (diluted in wash buffer), washed again, and the antibody-labelled proteins were visualized by enhanced chemiluminescence (Amersham Biosciences, Buckinghamshire, UK). Densitometric analysis was performed by normalising band density to that for β-actin using Quantity One v4.6.1 software (Bio-Rad, Hertfordshire, UK).

**Image Analysis**

Digital micrographs were obtained using a Nikon Eclipse 80i microscope (Nikon UK Ltd, Surrey, UK) equipped with a Qimaging digital camera (Media Cybernetics, Marlow, UK). At least 200 alveolar macrophages were counted per section and the number of immunopositive cells calculated and presented as a percentage of the macrophages population.

**Statistical analysis**

Data was analysed in GraphPad Prism and statistics performed in GraphPad Instat (GraphPad Software, San Diego, CA, USA; http://www.graphpad.com). ELISA data were treated parametrically and real time PCR data non-parametrically following normality tests.

Differences between groups was analysed using one-way ANOVA at each concentration of 17-BMP. Where there was statistical significance further unpaired students t-tests were performed between each group. Differences between 17-BMP and dexamethasone were analysed using paired t-tests at each concentration. EC50’s and IC50’s were generated using the mean data at each concentration. Real time PCR data was non-parametrically distributed. Friedman tests (non-parametric repeated measures ANOVA) were performed to analyse changes in gene expression within each group. Where there was statistical significance Dunn’s multiple comparison tests were performed. Two-tailed Mann-Whitney tests were performed between patient groups at each drug concentration. P<0.05 was considered significant. GR translocation data compound potencies, EC50 (effective concentration) and 95% confidence limits (CL), were derived from a four-parameter non-linear iterative curve fitting analysis.
